# Control of Polymers’ Amorphous-crystalline Transition for Hydrogel Bioelectronics Miniaturization and Multifunctional Integration

**DOI:** 10.21203/rs.3.rs-2864872/v1

**Published:** 2023-05-09

**Authors:** Sizhe Huang, Xinyue Liu, Shaoting Lin, Christopher Glynn, Kayla Felix, Atharva Sahasrabudhe, Collin Maley, Jingyi Xu, Weixuan Chen, Eunji Hong, Alfred J. Crosby, Qianbin Wang, Siyuan Rao

**Affiliations:** 1. Department of Biomedical Engineering, University of Massachusetts, Amherst, MA 01003, United States; 2. Department of Mechanical Engineering, Massachusetts Institute of Technology, Cambridge, MA, 02139, United States; 3. Department of Mechanical Engineering, Michigan State University, MI, 48824, United States; 4. Research Laboratory of Electronics, Massachusetts Institute of Technology, Cambridge, MA, 02139, United States; 5. Department of Polymer Science and Engineering, University of Massachusetts, Amherst, MA 01003, United States; 6. Institute for Applied Life Sciences, University of Massachusetts, Amherst, MA 01003, United States; 7. Neuroscience and Behavior Graduate Program, University of Massachusetts, Amherst, MA 01003, United States

## Abstract

Bioelectronic devices made of soft elastic materials exhibit motion-adaptive properties suitable for brain-machine interfaces and for investigating complex neural circuits. While two-dimensional microfabrication strategies enable miniaturizing devices to access delicate nerve structures, creating 3D architecture for expansive implementation requires more accessible and scalable manufacturing approaches. Here we present a fabrication strategy through the control of metamorphic polymers’ amorphous-crystalline transition (COMPACT), for hydrogel bioelectronics with miniaturized fiber shape and multifunctional interrogation of neural circuits. By introducing multiple cross-linkers, acidification treatment, and oriented polymeric crystalline growth under deformation, we observed about an 80% diameter decrease in chemically cross-linked polyvinyl alcohol (PVA) hydrogel fibers, stably maintained in a fully hydrated state. We revealed that the addition of cross-linkers and acidification facilitated the oriented polymetric crystalline growth under mechanical stretching, which contributed to the desired hydrogel fiber diameter decrease. Our approach enabled the control of hydrogels’ properties, including refractive index (RI 1.37-1.40 at 480 nm), light transmission (> 96%), stretchability (95% - 111%), and elastic modulus (10-63 MPa). To exploit these properties, we fabricated step-index hydrogel optical probes with contrasting RIs and applied them in optogenetics and photometric recordings in the mouse brain region of the ventral tegmental area (VTA) with concurrent social behavioral assessment. To extend COMPACT hydrogel multifunctional scaffolds to assimilate conductive nanomaterials and integrate multiple components of optical waveguide and electrodes, we developed carbon nanotubes (CNTs)-PVA hydrogel microelectrodes for hindlimb muscle electromyographic and brain electrophysiological recordings of light-triggered neural activities in transgenic mice expressing Channelrhodopsin-2 (ChR2).

Soft and elastic bioelectronics enable multifunctional interrogation of cell function from single-cell to organ-level resolution while providing tissue-like interfaces. In dynamically moving *in vivo* environments, such soft bio-interfaces can adapt to the persistent mechanical deformations of the living tissues, and consequently provide chronic, reliable access to biological systems. For the sophisticated yet delicate nervous system interfaces, elastic polymer materials, including polydimethylsiloxane (PDMS)^1^, cyclic olefin copolymer elastomer (COCE)^2^, polyurethane (PU) ^3^, alginate hydrogels^4,5^, have been deployed as the suitably elastic substrate for multifunctional devices that enable neural optogenetics stimulation^1,6,7^, electrophysiological recording^8,9^, drug infusion^10^ and neurotransmitter detection^11^. However, fabricating dedicated microstructures in soft and elastic devices is limited to 2D architectures and heavily relies on successive and sophisticated manufacturing approaches such as lithography^12,13^ and micro-printing ^14^.

Thermal pulling yields multiple-step scaling-down feasibility for multifunctional polymer fibers^10,15^; however, this approach requires coherent parameters of the constituent materials, such as glass transition temperature ($T_{g}$), melting temperature ($T_{m}$) and thermal expansion coefficients (α) to be drawn into an integrated fiber. Moreover, the high-temperature process narrows the selections of available polymers for high-water-content bioelectronics. Assisted with hydrogel cross-linking as a soft material matrix, hybrid multifunction fibers permit adaptive bending stiffness for long-term sensing and neural modulation^4,16^.

Besides mechanical stiffness change in the hydrated state and the desiccated state, hydrogel materials permit tunable volumetric control as the supporting scaffold. Employing hydrogel swelling behaviors in the solvated state, the expansion microscopy technique utilized hydrogel volumetric increase to enhance microimaging resolution for intact biological tissues^17^. In contrast, hydrogel shrinking behaviors in a desiccated state have been applied to densify patterned materials in volumetric scaffold deposition and obtain nanoscale feature sizes in three dimensions^18,19^. However, the hydrogel swelling and shrinking behaviors in these techniques are based on reversible polymer chains collapse in the desiccated state and expansion upon hydration. When applied to an aqueous in vivo environment, the shrunk hydrogels will expand and lose the miniaturized structures from the original manufacturing.

Inspired by the volumetric change resulting from polymer chains' folding and expansion, we hypothesize that control of the amorphous-crystalline transition in semi-crystalline hydrogels can enable intervention in polymer chain folding and crystallization. Consequently, this process prevents polymer chains’ expansion from their designed nanocrystalline structure in order to maintain hydrogels’ volumes under a solvated state. Hydrogel bioelectronics, miniaturized by the polymeric crystallization approaches, can stably maintain their designed architectures in vivo.

Here, we developed a set of cross-linking chemistry and micro-fabrication processes to control polymeric crystalline domain growth with cross-linked polyvinyl alcohol (PVA) hydrogels. A stable and tunable volumetric decrease of hydrogels was consistently achieved in a hydrated state under physiological conditions (pH 6-8, 37 °C). Through acidification treatment that increases polymer chain mobility while introducing dual cross-linkers of the inorganic binder tetraethyl orthosilicate (TEOS) and the generic glutaraldehyde (GA), we minimized the polymetric crystalline scattering (crystal size around 3.5 nm) and increased the hydrogels’ refractive indices (RI). Further nanocrystalline orientation induced by uniaxial deformation promoted the generation of nanoscale anisotropic architectures. This control of metamorphic polymers’ amorphous-crystalline transition (COMPACT) strategy enabled a 79.7% diameter decrease of hydrogel fibers in the hydrated state while maintaining high stretchability (94.5% - 111.2%) and low elastic moduli (9.7-62.5 MPa). Since COMPACT hydrogels provide a variety of RI options, we developed core-cladding hydrogel fibers with distinct RI contrast (n_core_=1.40, n_cladding_=1.34). These core-cladding structured hydrogel fibers were applied for concurrent photometry recordings from mouse brain ventral tegmental area (VTA) in the context of social interactions. Taking advantage of these tunable hydrogel matrix scaffolds, we loaded conductive nanomaterials, carbon nanotubes, into COMPACT hydrogels for hybrid microelectrodes. Integrated with an optical core, we produced multifunctional hydrogel optoelectronic devices for in vivo electrophysiological recording of optically triggered neural activities.

## Results

### COMPACT strategy for hydrogels controllable shrinking

**Figure 1.**
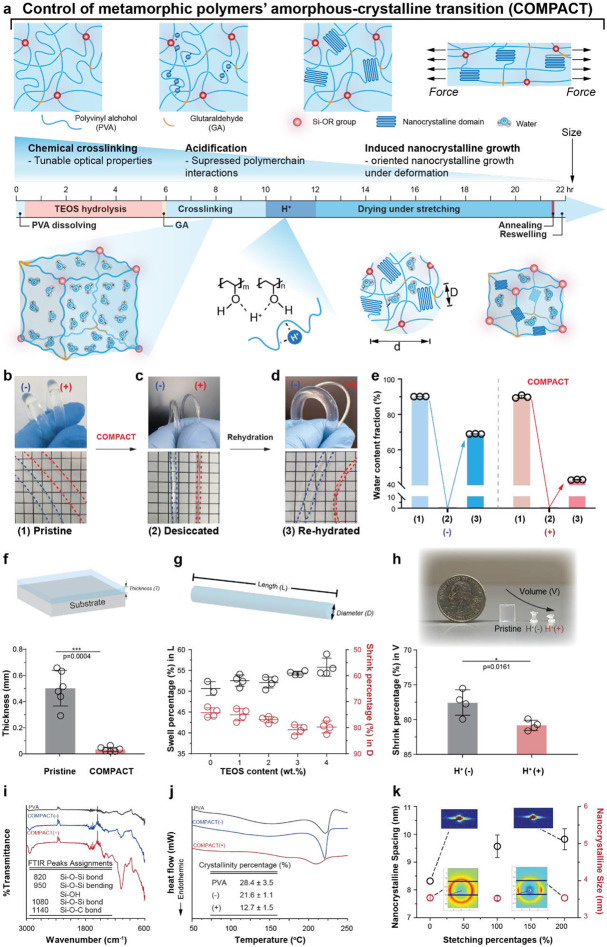
COMPACT strategy for hydrogel materials miniaturization. **a**, Schematic illustrations of hydrogel network of metamorphic polymers’ amorphous-crystal transition (COMPACT). COMPACT treatment includes cross-linking with both glutaraldehyde (GA) and tetraethyl orthosilicate (TEOS), acidification and mechanical stretching. **b-e**, Representative photographs and water contents of TEOS-GA cross-linked polyvinyl alcohol (PVA) hydrogel with COMPACT treatment (+) and GA cross-linked hydrogel without acidification and stretching (−) at the pristine state (**b**), desiccated state (**c**) and re-hydrated state (**d**). Grid size: 5 mm. **f**, Shrinking behaviors of TEOS-GA cross-linked PVA (4 wt.% TEOS) hydrogel film with acidification treatment. Film thickness is quantified as mean ± standard deviation (s.d., paired student’s t-test, ***p=0.0004). Each dot represents one individual film. **g**, Shrinking behaviors of COMPACT hydrogel fibers (1-4 wt.% TEOS and 200% stretching). Hydrogel fibers’ length (black) and diameter (red) are quantified as mean ± s.d. Each dot represents one independent fiber. **h**, Shrinking behaviors of cross-linked hydrogel cylinders. The volume of TEOS-GA cross-linked hydrogel cylinders (4 wt.% TEOS) and with acidification treatment and GA cross-linked hydrogel cylinders without acidification treatment are compared with mean ± s.d. (unpaired student's t-test, F3,3=6.084, *p=0.0161). Each dot represents one independent hydrogel cylinder. **i**, Fourier transform infrared (FTIR) spectroscopy of COMPACT (−) and COMPACT (+) hydrogels. **j**, Differential scanning calorimetry (DSC) profiles of COMPACT (−) and COMPACT (+) and their crystallinity percentages. **k**, Small-angle X-ray (SAXS) and wide-angle X-ray (WAXS) results of hydrogel materials in the desiccated state (mean ± s.d.). Inset: SAXS and WAXS 2D patterns.

Chemically cross-linked PVA hydrogels have been widely employed with superior optical properties^20^, fatigue-resistance^21, 22^, and biocompatibility for bioelectronics applications^23, 24^. To further explore PVA hydrogels’ controllable miniaturization properties while preserving these advantageous features, we designed new hydrogels fabrication approaches by control of metamorphic polymers’ amorphous-crystalline transition (COMPACT) with the following aspects: (i) polymer chains folding and immobilization with multiple cross-linkers, (ii) intervention on intermolecular chain interactions in the hydrogel matrix, (iii) inducing the oriented growth of nanocrystalline domains. We implemented the COMPACT strategy following three major procedures to control individual polymer chain folding, polymer chain network interactions and nanocrystalline growth. We first introduced the hydrolysis of TEOS in PVA solutions through homogenization (Fig. 1a and **Supplementary Note 1**), followed by the addition of a generic cross-linker, GA. A combination of two types of cross-linkers is chosen to allow the control of polymer chain mobility via covalent bonding and parallel tuning of hydrogels’ refractive index. We then acidified the cross-linked hydrogels to promote intermolecular chain interactions and to facilitate the formation of nanocrystalline domains in hydrogels. External mechanical stretching was applied to the fully acidified hydrogels and maintained during the desiccating process. After the removal of water molecules from hydrogels, high-temperature (100 °C) annealing was employed to further promote the growth and orientation of the nanocrystalline domains. To test whether polymeric nanocrystalline domains created through the COMPACT strategy can preserve hydrogels volumetric shrinking under hydrated status, we next examined the dimensions and water fractions of cross-linked hydrogels under pristine, desiccated, and re-hydrated states (Fig. 1b-e).

We prepared fiber-shaped hydrogels via molding and extrusion methods (**Supplementary Note 2**). At the pristine (Fig. 1b) and desiccated states (Fig. 1c), the two hydrogel fibers with TEOS-GA cross-linking (COMPACT+) and GA cross-linking (COMPACT−) exhibited comparable geometries and water fractions (Fig. 1e); however, only the TEOS-GA cross-linked PVA hydrogel fiber with acidification and mechanical stretching maintained the reduced diameters in the rehydrated state (Fig. 1d, e).

After we confirmed that hydrogels retained shrinking behaviors in the re-hydrated state with COMPACT treatment, we tested whether size reduction is dependent on the materials' geometries and external constraints. We prepared hydrogels with the shapes of thin film, fiber, and block, and examined the changes of COMPACT hydrogel film thickness (*T*, Fig. 1f), fiber diameter (*D*, Fig. 1g) and volume (*V,* Fig. 1h). TEOS-GA cross-linked PVA hydrogel thin films with acidification treatment exhibited a thickness reduction ratio of 93.4 ± 3.6% (pristine thickness: 501 ± 134 μm; re-hydrated thickness: 33 ± 18 μm) under optical microscopy examination (Fig. 1f). TEOS-GA cross-linked PVA hydrogel fibers, with applied acidification and mechanical strain (200%) treatments, reached the maximum diameter shrinking ratio of 79.7 ± 2.3%, by increasing the content of the TEOS cross-linker (Fig. 1g). In three-dimensional free shrinking structures, we observed 80.9± 0.7% volumetric shrinking in acidified TEOS-GA cross-linked cylinders as compared to pristine ones (Fig. 1h).

We then investigated the mechanisms of the sustained hydrogel volume decrease and the design of amorphous and crystalline architectures. Fourier transform infrared spectroscopy (FTIR) results indicated covalent bonds (Si-O-Si and Si-OH) generated in the COMPACT hydrogel network (Fig. 1i). The new Si-O-Si (1080 cm^−1^) and Si-OH (950 cm^−1^) bonds came from hydrolyzed TEOS Si-OR groups’ reactions with the hydroxyl groups on PVA chains. The generic cross-linker GA reactions were confirmed by the observation of C=O bond (1740 cm^−1^) and the Si-O-C bond (1140 cm^−1^) from the reaction with TEOS Si-OR groups. Besides confirming covalent bonds generated among hydrogel polymer chains, differential scanning calorimetry (DSC) results exhibited the change of polymer chain interactions and polymeric crystallinity after COMPACT treatment. Undissolved PVA powders showed 28.4 ± 3.5% crystallinity (Fig. 1j and **Supplementary Fig.2**), similar to the reported crystallinity percentage of semi-crystalline PVA polymers^25^. GA-cross-linked PVA hydrogels exhibited 21.6 ± 1.1% crystallinity while the additional TEOS cross-linking, and acidification suppressed the polymer chain folding to form crystalline domains (crystallinity: 12.7 ± 1.5%). We further examined the nanocrystalline domains and orientation with X-ray scattering techniques. The size of PVA nanocrystals was measured as 3.5 ± 0.1 nm while the nanocrystalline spacing increased from 8.4 nm to 10.2 nm after 200% axial stretching (Fig. 1k and **Supplementary Fig. 2-4**). Wide-angle X-ray scattering (WAXS) 2D patterns suggested that the lamellae crystal domains were re-oriented along the axial stretching direction (Fig. 1k and **Supplementary Fig. 4**).

### COMPACT hydrogel fibers’ tunable properties

**Figure 2.**
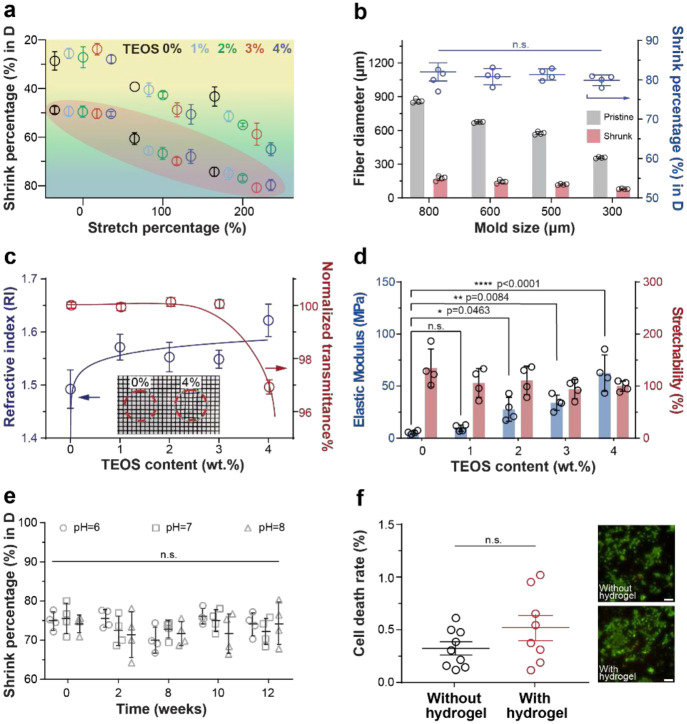
Controllable hydrogel fiber fabrication and its properties. **a**, A shrinking diagram of COMPACT (+) hydrogel fibers. Each dot (mean ± s.d.) represents an independent hydrogel fiber sample. The samples shaded in red areas are treated with acidification. **b**, Shrinking behaviors of COMPACT hydrogel fibers (4wt.% TEOS) prepared in different sizes of molds. Each dot (mean ± s.d.) represents one independent fiber (one(One-way ANOVA and Tukey's multiple comparisons test, F_3,12_=0.9543, n.s.: not significant. p=0.4455). **c**, COMPACT hydrogel fibers’ optical properties of refractive index (blue) and normalized light transmittance (red) (mean ± s.d). Inset: representative photographs of 0 wt.% TEOS and 4 wt.% TEOS hydrogel membranes. Grid size: 1 mm. **d**, COMPACT hydrogel fibers’ mechanical properties of elastic modulus (blue) and stretchability percentage (red). Each dot represents one independent fiber sample. One-way ANOVA and Tukey's multiple comparisons test were used to determine the statistical significance of elastic modulus: (F_4,15_=20.51, ****p<0.0001;) and stretchability: (F_4,15_=1.492, n.s. p=0.2543), respectively. **e**, Stability assessment of diameter reduction of COMPACT hydrogel fibers (3wt.% TEOS). Each dot (mean ± s.d.) represents one independent fiber (two-way ANOVA and Tukey's multiple comparisons tests). **f**, Cytotoxicity assessment of COMPACT (+) hydrogels. Hydrogel incubated media was used to culture with HEK293 cell cultures. Calcein-AM (green) was used to stain living cells and ethidium homodimer-1 (red) was used to stain dead cells. Cell death rates are presented as mean ± standard error (s.e.m., unpaired student’s t-test).

With COMPACT-enabled hydrated hydrogel size reduction, we expanded this methodology to develop a series of hydrogel fibers with controlled diameters and tunable optical and mechanical properties for biomedical use. We mapped a rational and comprehensive shrinking diagram by varying the content of inorganic cross-linker (TEOS), acidification, and external mechanical stretching (Fig. 2a). Generally, increasing cross-linking density with more cross-linkers yielded less ductile polymer chains with reduced dimension upon hydration. Acidification treatment dramatically boosted shrinking percentages across different cross-linking densities while mechanical static stretching further decreased hydrogel fibers in diameters (79.7 ± 2.3%). To fit COMPACT into a practical molding-extrusion fabrication process (**Supplementary Note 2**)^26^, we examined a series of hydrogel fibers made with different sizes of silicone molds (Fig. 2b and **Supplementary Fig. 5**). Independent from the mold size, all COMPACT hydrogel fibers reached reduced diameters more than 79%, which is consistent with the shrinking diagram (Fig. 2a). As an example, using 300 μm (inner diameter, ID) silicone molds, thin hydrogel fibers were fabricated with diameters of 80 ± 4 μm.

Considering their fiber optic in vivo applications^27^, we examined the optical, mechanical and biocompatible properties of COMPACT hydrogel fibers. To ensure efficient light transmission for optical stimulation and recordings, we considered two important parameters of the hydrogel fiber core: refractive index (RI) and light transmittance. We observed that hydrogels’ refractive indices can be tuned by increasing TEOS contents. COMPACT hydrogels with 0 wt. *%* to 4 wt. *%* TEOS contents exhibited refractive indices ranging from 1.48 to 1.60 in the desiccated state (Fig. 2c) and 1.37 to 1.40 in the hydrated state (**Supplementary Fig. 6a-b)**, which is comparable with the RI of other conventional polymer hydrogels^28^. Although all the transmittance remained above 96%, increasing TEOS content also led to decreased transmittance (Fig. 2c and **Supplementary Fig. 6c**), and increased autofluorescence (17.8% increase of 4 wt.% TEOS hydrogels compared to 0 wt. % TEOS hydrogels, excitation wavelength: 485 nm, excitation peak: 520 nm, **Supplementary Fig. 6d)**. The optimal TEOS content was chosen as 3 wt. %, which resulted in hydrogels with 1.54 ± 0.01 of refractive index (Fig. 2c), > 96% of transmittance ((Fig. 2c, for 0.15 ± 0.02 mm thick membranes), and 6.13 ± 0.16 relative fluorescent units (RFU)/mm of autofluorescence (for 0.15 ± 0.02 mm thick membranes. water: 3.70 RFU/mm, **Supplementary Fig. 6d**).

We then examined whether COMPACT hydrogels maintained tissue-like elasticity. COMPACT hydrogel fibers exhibited relatively low elastic moduli while maintaining high stretchability (Fig. 2d and **Supplementray Fig. 7a-b**). The optimized COMPACT hydrogel fiber (3 wt.% TEOS, 12 mM HCl acidification treatment and 200% stretching, diameter: 227 ± 18 μm) exhibited an elastic modulus of 34.03 ± 7.38 MPa. Compared to silica fibers (~20GPa elastic modulus)^29^ and polymer fibers (~1GPa elastic modulus)^2,4^, COMPACT hydrogel fibers offer enhanced mechanical matching to the nervous tissues (1-4 kPa)^30^ and lead to less neural tissue damage from micro-motion involved in vivo studies^31^.

We then tested whether crystalline-enabled size reduction of COMPACT hydrogels can overcome the intrinsic hydrogel swelling exhibited upon hydration and maintain structural stability in vivo, we incubated COMPACT hydrogel fibers in ex vivo physiological conditions (pH: 6-8, 37 °C, saline solution) and monitored fibers’ dimension over time. We observed the shrinking percentage maintained above 74% over 3 months (Fig. 2e and **Supplementary Fig. 8**). Cytotoxicity tests with human embryonic kidney cells (HEK293) exhibited no significant cell death in the presence of COMPACT hydrogels (Fig. 2f and **Supplementary Fig. 9**).

### Step-index hydrogel optical fibers

**Figure 3.**
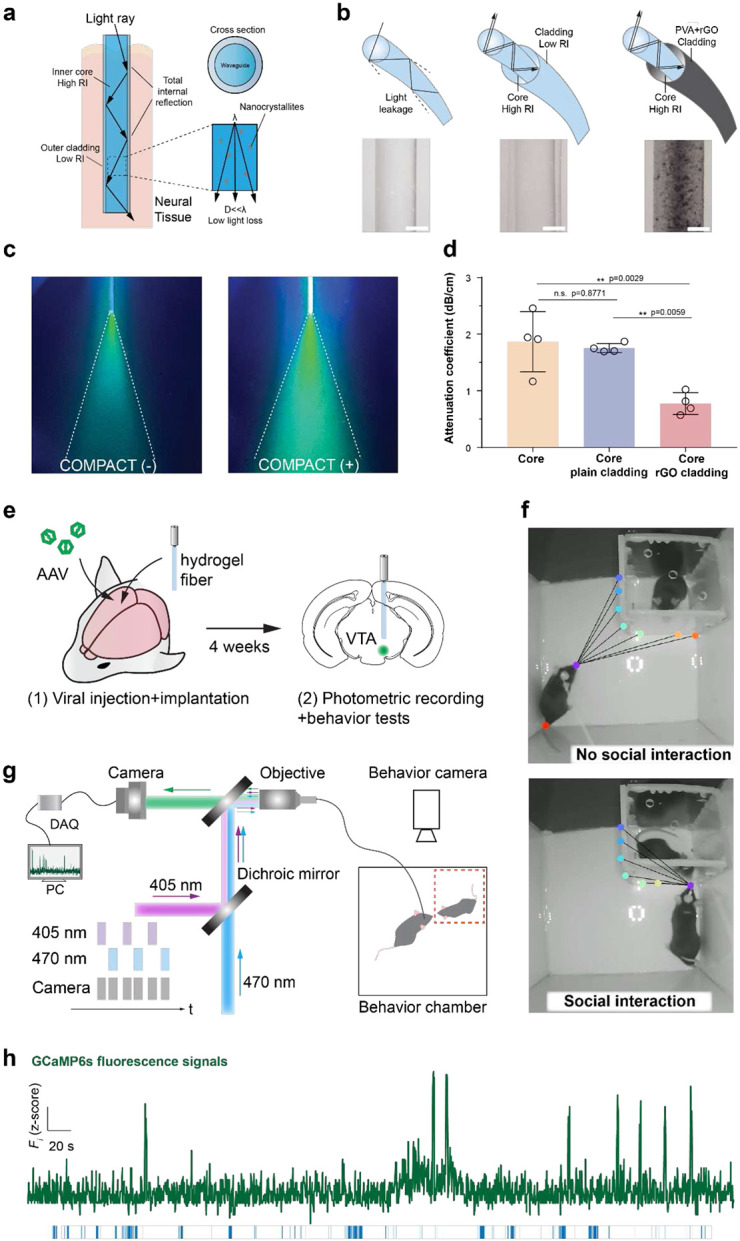
Hydrogel optical neural probes for photometric recording with behavioral assessment. **a**, A schematic illustration of light transmission in a step-index hydrogel fiber. **b**, Schematic illustrations and representative photographs of a COMPACT core hydrogel fiber, a COMPACT core-plain-cladding hydrogel fiber, and a COMPACT core-rGO-cladding fiber. Scale: 200 μm. **c**, Representative photographs of blue light (480 nm) transmission from a COMPACT (−) core hydrogel fiber and a COMPACT (+) core hydrogel fiber into solutions containing Calcein fluorescent dye. **d**, Light attenuation coefficients of COMPACT core hydrogel fibers, COMPACT core-plain-cladding hydrogel fibers, and COMPACT core-rGO-cladding fibers (mean ± s.d., one-way ANOVA and Tukey's multiple comparisons test, F_2,9_=13.3, **p=0.0021). Each dot presents one independent hydrogel fiber sample. **e,** Experimental scheme for the viral injection, optical fiber implantation, photometric recording and social behavior tests. **f,** Representative images in mouse social interaction tests. **g,** A schematic illustration of fiber photometry recording setup with concurrent mouse social behavior tests. **h,** Normalized fluorescence intensity change (ΔF/F_0_) of GCaMP6s in the VTA from mice social interactions. Blue bars indicate social interaction time.

COMPACT hydrogels were first fabricated into step-index optical fibers (**Supplementary Note 3**). Increased RI contrast between optical core and cladding layers ensures light transmission and the consequent photodetection sensitivity (Fig. 3a). Based on tunable refractive indices of COMPACT hydrogels (Fig. 2c and **Supplementary Fig. 6 a-b**), we designed step-index hydrogel fibers with high-RI core (n_core_=1.40) and low-RI cladding (n_cladding_=1.34).

Hydrogel fibers were connected to a silica segment embedded in an optical ferrule, which provides a strong connection while preventing directly exposed hydrogel dehydration out of tissues and light loss (**Supplementary Note 3**). We validated the function of RI-contrasting core-cladding structures by comparing the light transmission between bare core fibers, step-index fibers with plain cladding and those with light-protective cladding (Fig. 3b-c, and **Supplementary Note 3-4**). The bare core fibers (diameter of 329 ± 17 μm) exhibited a relatively high attenuation (1.87 ± 0.53 dB/cm) while introducing a thin low-RI cladding layer (thickness of 84 ± 4 μm on the surface of 372 ± 10 μm cores, n_cladding_=1.34) decreased the light transmission attenuation to 1.75 ± 0.08 dB/cm (Fig. 3c). A representative light-absorption nanomaterial^32,33^, reduced graphene oxide (rGO) was loaded into low-RI cladding to further protect light leakage from fibers’ lateral surface and consequently reduced the light attenuation to 0.94 ± 0.25 dB/cm (core 339 ± 35 μm, cladding: 36 ± 11 μm of 5 wt.% PVA with 0.21 wt.% rGO) (Fig. 3c and **Supplementary Fig. 10**).

To validate their functionality for in vivo optical interrogation, we tested COMPACT hydrogel fibers with fiber photometric recording in the context of mouse social behaviors. Activation of VTA region and its related circuits has been studied with various techniques, including optogenetics^34^, electrical stimulation and chemogenctics^35^, related to social behaviors in mice^36^. As a proof-of-concept application, we applied COMPACT hydrogel fibers to record mouse deep brain structure, VTA, with concurrent social behavior observation. We unilaterally implanted COMPACT optical fibers (580 ± 35 μm) in VTA after injecting of adeno-associated virus (AAV) containing genetically encoded calcium indicator (*hSyn*::GCaMP6s) (Fig. 3e). A home-built fiber photometry system (wavelengths: λ_isosbestic point_=405 nm, λ_excitation_=470 nm, λ_emission_=510 nm) based on the previous design was used to collect GCaMP fluorescent change as a proxy to reflect the neural activity^37^ (Fig. 3g and **Supplementary Fig. 13**). We utilized the stiffness change of hydrogel fibers from a desiccated state (stiff) to a hydrated state (soft) and implanted the hydrogel fiber in the desiccated state with calibrated coordinates (**Supplementary Figure. 11-12**). After an incubation period of 4 weeks for AAV virus expression, we subjected mice to a social behavioral test with concurrent photometric recordings. Mouse social interactions were analyzed with DeepLabCut markless pose estimation and a custom-developed MatLab algorithm (Fig. 3f). We found that increased fluorescent intensity of GCaMP was correlated with mouse social interaction epochs (Fig. 3h).

### COMPACT multifunctional hydrogel neural probes

**Figure 4.**
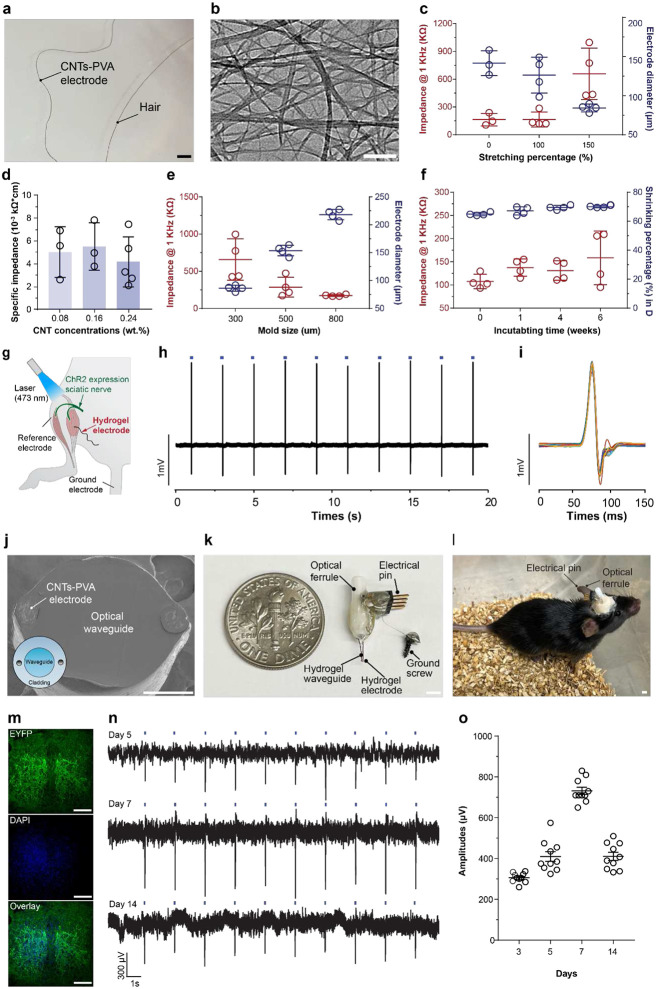
Integrated multifunctional hydrogel neural probes. **a**, A Representative photograph of a carbon nanotube (CNT)-PVA hydrogel electrode as compared with a piece of human hair. Scale: 300 μm. **b**, A transmission electron microscopy (TEM) image of CNTs. Scale: 200 nm. **c**, Impedance at 1 kHz (red dots) and diameters of the electrodes (blue dots) fabricated different stretching percentages (mean ± s.d.). Each dot represents one independent hydrogel electrode. **d**, Impedance at 1 kHz of electrodes fabricated with different CNT concentrations (mean ± s.d.). Each dot represents one independent hydrogel electrode. **e**, Impedance at 1 kHz of electrodes (red dots) and diameters of the electrode (blue dots) fabricated with different sizes of molds (mean ± s.d.). Each dot represents one independent hydrogel electrode. **f**, Stability assessment on impedance (red dots) and diameters (blue dots) of hydrogel electrodes (mean ± s.d.). Each dot represents one independent hydrogel electrode. **g,** A schematic illustration of electrical recordings from mouse gastrocnemius muscles with a CNTs-PVA electrode in the presence of transdermal optical stimulation. **h,** Representative EMG signals recorded with CNT-PVA hydrogel electrodes upon transdermal optogenetic stimulations in *Thy1::ChR2-EYFP* mice (λ=473 nm, 0.5 Hz, pulse width 50 ms, 200 mW/mm^2^). Blue bars indicate the light illumination periods. **i**, Overlay plot of EMG peaks. **j**, A scanning electron microscopy (SEM) image at the cross-section of an integrated multifunctional neural probe containing an optical core and two CNT-PVA hydrogel electrodes. Scale: 100 μm. **k-l**, Photographs of a hydrogel optoelectronic device (optrode) before implantation and after implantation in a *Thy1::ChR2-EYFP* mouse brain. Scale: 2 mm. **m**, Confocal images of the expression of ChR2-EYFP in the VTA region of mouse. Scale: 50 μm. **n**, Representative in vivo electrophysiological signals recorded with optrodes upon optical stimulation (blue bars, λ=473 nm, 0.5 Hz, pulse width 50 ms, 10 mW/mm^2^). **l**, Amplitudes of electrophysiological signals recorded with optical stimulation on day 3, day 5, day 7, and day 14 post-implantation (λ=473 nm, 0.5 Hz, pulse width 50 ms, 10 mW/mm^2^, mean ± s.e.m.).

Hydrogel matrix can support various nanoscale materials to extend the functionalities while maintaining desired mechanical properties^35,38^. To enrich hydrogel neural probes’ modality for electrical recordings, we incorporated conductive carbon nanotubes (CNTs, 12 ± 6 nm diameter) into PVA hydrogel scaffolds during hydrogel cross-linking (Fig. 4a and **Supplementary Note 5**). Acidification and mechanical stretching facilitated CNT plaiting into polymer matrices and ensured entanglement with PVA chains and consequently augmented electrical conductivity as a percolated network^39,40^. CNTs-PVA hydrogel electrodes (86 ± 5 μm diameter) exhibited stable impedances of 658 ± 277 kΩ at 1kHz (PBS, 25 °C, Fig. 4c and **Supplementary Fig. 15**) and impedance was tunable with designed mold sizes and CNT loadings (Fig. 4d and f). CNTs-PVA hydrogel electrodes were insulated with a viscoelastic coating of styrene-ethylene-butylene-styrene (SEBS) (**Supplementary Note 5** and **Supplementary Fig. 16**). To verify the stability of CNTs-PVA hydrogel electrodes, we incubated them in PBS solutions and characterized the impedance over 6 weeks (Fig. 4e). No significant increase of impedance at 1kHz was found.

Then we deployed CNT-PVA hydrogel electrodes for electromyographic (EMG) recordings of mouse hindlimb muscles in response to the pulsed blue light illumination. CNT-PVA hydrogel electrodes detected hindlimb muscle electrical signals upon transdermal optical stimulation (wavelength λ=473 nm, 200 mW/mm^2^, 0.5 Hz, pulse width 50ms) in *Thy1::ChR2-EYFP* mice, which express photo-excitatory opsin, Channelrhodopsin 2 (ChR2), in the nervous system (Fig. 4f). EMG signals exhibited repeatable amplitude and signal-to-noise ratios, which indicates the reliability of CNT-PVA hydrogel microelectrodes.

When extending hydrogel miniaturization from bulk materials to interfaces, the COMPACT strategy offers a new avenue for multiple components integration. Since RI-distinct core-cladding structures ensure light transmission in optical cores, we introduced two CNT-PVA electrodes into the cladding layers with a COMPACT hydrogel core (Fig. 4b). A hydrogel optoelectrical device (optrode), is designed to enable optical modulation with simultaneous electrophysiological recording (**Supplementary Note 6)**. In *Thy1::ChR2-EYFP* mice, blue light pulses (λ=473 nm, 0.5 Hz, pulse width 50 ms, 10 mW/mm^2^), delivered through the hydrogel optical core, consistently activated ChR2-expressing neurons in VTA while the neural electrical signals were collected through CNT-PVA electrodes (Fig. 4l). The optical evoked potentials were repeatedly captured with correlation with the onset of light stimulation over two weeks post-implantation.

## Discussion

In this study, we developed a set of hydrogel cross-linking chemistry and fiber-shaped device microfabrication approaches through a bottom-up strategy of tuning polymers’ amorphous-crystalline transition for hydrogel bioelectronics miniaturization and integration. COMPACT provides an accessible, scalable, and controllable fabrication method for micro-structured hydrogel fibers as small as 80 μm with consistently low asperity. These hydrogels provide a platform for functionally augmented interfaces through loadings of additional nanomaterials. COMPACT hydrogels can be further designed into step-index optical probes and optoelectronic devices (optrodes) which are well-suited for neural modulation and recordings concurrent with behavioral assays in mice.

Unlike established approaches to shrink hydrogels via desiccation, where collapse of polymer chain during drying leads to reversible swelling upon hydration, COMPACT hydrogels’ polymetric nanocrystalline and enhanced interpolymer chain interactions maintained stable folding in the hydrated state and therefore permit retained volumetric size reduction. Over 3 months of incubations under physiological temperature and osmolarity, the shrunk COMPACT hydrogel fibers maintained the designed diameters within less than 1% variance (Fig. 2e), which illustrates COMPACT bioelectronics’ volumetric stability of their miniaturized size in vivo. In contrast, COMPACT hydrogel fibers incubated at PVA dissolution temperature (100 °C) in water for several hours resumed their pristine swollen size; this volume reversion demonstrates the crystalline impact on size reduction through control of local free volume in hydrogel matrices. This crystalline-dominated hydrogel miniaturization phenomenon can be extended to other semi-crystalline polymers at different material interfaces, where volumetric stability is important, such as the proton-exchange membrane in packed fuel cells.

In COMPACT hydrogels, chemical cross-linkers and acidification treatment both contribute to the retained volumetric decrease upon re-hydration while mechanical deformation induced the orientated nanocrystalline growth. An increased number of chemical cross-linkers, TEOS (0 wt.% to 4 wt.%, Fig. 1g), enhanced the anchoring of amorphous PVA chains through covalent cross-linking and prevent swelling in the hydrated state. Under the same cross-linking degree, acidification treatment granted polymer chains enhanced interactions and suppressed crystallinity (Fig. 1j and **Supplementary Fig. 1 and 7c**). Nanocrystalline domains maintained the nanoscale size (~3.5 nm) without compromising the transmittance in the visible range. Axial mechanical deformation re-orientated nanocrystalline and created anisotropic nanostructures (Fig. 1k), which enabled hydrogel fibers’ desired decrease in diameter while causing a minimal effect on crystallinity degree (**Supplementary Fig. 1c**) or nanocrystalline size (Fig. 1k).

Controllable hydrogel shrinking provides an effective methodology for miniaturization and integration for neural probe fabrication. The molding and extrusion approaches offer a series of precisely controlled hydrogel fiber diameters with structural homogeneity and low surface asperity to avoid diffuse reflection at the hydrogel interfaces. COMPACT hydrogel fibers’ tissue-like mechanical properties exhibit improved immune response compared to stiff silica fibers (Fig. 2d and **Supplementary Fig. 14**). Although the mold sizes are commercially limited, COMPACT procedures, including regulating polymer and crosslinker constituent content and fiber extensions can expand the range of available fiber sizes. Successive rounds of molding with strong polymer chain infiltration at the interfaces enable the design of multimodal microstructures, including core-cladding (30-80 μm) in step-index optical probes and electrode integration in the cladding layer of optrodes. Currently, the number of integrated components, such as electrodes and microfluidic channels, is limited by the coaxial alignment in the secondary molding step; the accessibility and throughput of multimodal fabrication can be further improved with guiding devices to facilitate integration and alignment, or alternative coating approaches.

COMPACT strategy is generalizable for soft and stretchable bioelectronics. Polymer matrices provide sufficient free volume for water access as well as nanomaterials’ incorporation. High aspect-ratio nanomaterials, such as silver nanowires and carbon nanotubes, can be effectively entangled with polymer chains through cross-linking and condensation during acidification and stretching. This procedure augments electrical conductivity while maintaining viscoelasticity. The colloidal stability of nanomaterials in viscous polymer precursor solutions is important to create a homogeneous composite after cross-linking to prevent phase separation and ensure stable electrical conductivity.

Compared to other soft bioelectronics fabrication approaches, such as lithography and micro-printing, COMPACT technique offers scalable and efficient multimodal hydrogel fibers manufacturing without the need for expensive and sophisticated facilities. COMPACT multifunctional neural probes have been employed for bi-directional optical interrogation concomitant with mouse social behaviors and electrical recordings of light-triggered neural activity in mice. Extended functionalities, such as drug or viral vector delivery, can be further achieved by integrating additional microfluidic channels in the cladding layer and retains light transmission efficiency in the optical core. COMPACT multifunctional neural probes involve independent components alignment and miniaturization steps, which potentiates the integration of multiple components with various lengths to target multiple depths of tissue within single-step implantation. This adaptability will increase the density of functional interfaces and overcome the traditional limitation of fiber-shaped neural probes with single-target interfaces at the tip.

Control over semi-crystalline polymers’ amorphous-crystalline transition creates a direct fabrication methodology for elastic soft materials. Extending it to the manufacture of sophisticated optoelectronic devices, the COMPACT strategy imparts a generalizable and modular platform for hydrogel bioelectronics’ miniaturization and integration, which consequently enables multimodal interrogation of complex biological systems.

## Methods

### Hydrogel synthesis.

The chemicals used in this study included tetraethyl orthosilicate (TEOS, Sigma-Aldrich 86578, 99%), hydrochloric acid (HCl, Sigma-Aldrich, 258148, 37%), glutaraldehyde solution (GA, Sigma-Aldrich G6257, 25% in water), and polyvinyl alcohol (PVA) with an average molecular weight of 146,000 to 186,000 Da and 99+% hydrolyzed (Sigma-Aldrich, 363065). MilliQ water with a resistivity of 18 MΩ·cm at 25 °C was used throughout the experiments. To prepare the PVA (10 wt. %) solution, PVA was dissolved in MilliQ water and stirred in a water bath at 100 °C for at least 4 hours until a clear and transparent solution was obtained. The hydrolysis of TEOS was carried out using HCl as a catalyst in PVA solutions with a molar ratio of TEOS: HCl: H2O=x: 4: y, where x was between 1 to 4, and y started from 4 to 16. TEOS solutions with concentrations ranging from 2 wt.% to 8 wt.% were added to the PVA solutions, which were then homogenized at two different levels. A mixture of HCl and MilliQ water in a molar ratio of 4: y, where y was in the range of 4 to 16, was added dropwise to the PVA-TEOS emulsion while homogenizing at 12000 rpm using a portable homogenizer until a stable emulsion was formed. The resulting emulsion was further homogenized using a high-speed homogenizer (FSH2A lab). The mixed solutions were stirred in a water bath at 100 °C for 1 hour until transparent solutions were obtained, followed by an additional 12 hours of stirring at 60 °C. The composition of all solutions used in this study is provided in Table 1.

### Optical hydrogel probe fabrication.

A step-index multimode silica fiber (core diameter 400 μm, NA 0.5, Thorlabs FP400URT) was prepared by removing the protective coating using a fiber stripping tool (Micro-strip, Micro Electronics, Inc). The stripped fiber was then divided into 13-mm segments using a diamond cutter. These fiber segments were inserted and extruded from one end of an optical ferrule (bore diameter 400 μm, Thorlabs CFX440-10) with a length of 2.5 mm and secured with EccoBond F adhesive (Loctite). Both ends of the silica fibers in the ferrules were polished using a polish kit (Thorlabs D50-F, NRS913A, and CTG913). The light transmission of all silica fibers and ferrules was tested by coupling with a 470 nm blue light-emitting diode (LED) (Thorlabs M470F3) after polishing. To remove the plastic coatings on the extruded silica fibers, they were treated with 2M sodium hydroxide solution (Sigma-Aldrich, 1064980500) for 2 hours followed by an additional treatment with chloroform (Sigma-Aldrich, 472476) for 30 minutes. A thin layer of 10 wt.% PVA was then coated on the extruded silica fibers via dip coating, and the PVA-coated silica fibers were air-dried at room temperature for 12 hours and annealed at 100 °C for 2 hours. A vacuum planetary mixer (Musashi ARV-310, 2000 rpm, and 16 kPa vacuum) was utilized for the mixing and degassing of all solutions. For degassing and mixing, 100 μL of GA was added to 10 g of 10 wt.% PVA pre-solution and agitated for 1 minute. 10 g of pre-made PVA-TEOS solution was also degassed and mixed for 1 minute. Subsequently, the above two solutions were combined (weight ratio of 1:1) and mixed for another minute. The resulting PVA-TEOS-GA solution was infused into silicone tubes (McMaster-Carr 5236k204, 80 mm in length), and the optic ferrules were inserted into the silicone tubes, with the silica fiber end connected to the PVA mixture. After curing at room temperature for 4 hours, the PVA-TEOS-GA fibers were demolded using dichloromethane (DCM, Sigma-Aldrich, 270997, 99.8%) and washed with a large amount of water to remove residual chemicals for two days. Ferrule-connected fibers were air-dried at room temperature for 12 hours and annealed at 100 °C for 20 minutes. Finally, the hydrogel fibers were rehydrated with MilliQ water before use. The compositions of all fabricated fibers are listed in Table 2.

### Core-cladding optical probe fabrication.

A vacuum planetary mixer (Musashi ARV-310, 2000 rpm, and 16 kPa vacuum) was employed for mixing and degassing of all solutions. The optical fiber probes were first dried and then re-inserted into silicone tubing (McMaster-Carr 51845K66) and reswelled in water. For the preparation of the core-cladding optical fiber probes, 100 μL of GA was added to 10g of 5 wt.% PVA pre-solution, which was then degassed and mixed for 1 minute. Additionally, 150 μL of HCl was added to 10g of 5 wt.% PVA pre-solution, which was also degassed and mixed for 1 minute. The two solutions were combined (weight ratio of 1:1) and mixed for 1 minute. The resulting mixed solution was infused into the silicone tubing and allowed to cross-link for 4 hours at room temperature. The core-cladding optical fiber probes were extruded by immersing them in DCM and stored in MilliQ water until further use.

### XRD characterization of hydrogel materials.

X-ray scattering measurements were conducted using the SAXSLAB GANESHA 300XL instrument, equipped with a Dectris Pilatus 300K 2D CMOS photon counting detector (size: 83.8 x 106.5 mm^2^). A small-angle 2 mm beamstop was utilized for SAXS measurements, while a wide-angle 2 mm beamstop was employed for WAXS measurements. The exposure time was set at 600 seconds. The average size of the nanocrystalline domain was determined using Scherrer’s equation, which is expressed as $D=\frac{k\lambda }{\beta \;\cos,\theta }$, where k is a dimensionless shape factor that varies based on the actual shape of the nanocrystalline domain (k=1, approximating the spherical shape of the nanocrystalline domains), λ is the wavelength of X-ray diffraction (λ=1.54 Å), θ is the peak of the Bragg angle, and β is the full width at half maximum (FWHM) of the WAXS peaks. The d-spacing between nanocrystalline domains was calculated using $d=\frac{2\pi }{q_{\max}}$, where $q_{\max}$ is the q value at its maximum intensity from SAXS patterns. The FWHM (β) and $q_{\max}$ were obtained by curve fitting of the WAXS and SAXS patterns, respectively, in Origin software (OriginLab Corporation).

### DSC characterization of hydrogel materials.

The degree of crystallinity of hydrogel fibers and materials was assessed using a DSC instrument (2920 TA instrument). The PVA hydrogels were analyzed in the desiccated state. A small quantity of sample (1-15 mg) was loaded into a crucible (TA instrument T81006) and inserted into a temperature-controlled DSC cell. A blank crucible served as a reference. The sample was heated from 30 °C to 300 °C in air, with a heating rate of 20 °C/min. The differential heat flow to the sample and reference was recorded by the instrument. To determine the melting fusion enthalpy of endothermic peaks, heat flow (mW) over sample weight (mg) was plotted against time (s). The areas of melting endothermic peaks were integrated using TA analyze software (TA Universal Analysis). The degree of crystallinity α was estimated using the equation: $\alpha =\frac{\Delta H_{m}}{\Delta H_{m}}$. 100%, where $\Delta H_{m}$ (J/g) was calculated from the integration of melting endothermic peaks and $\Delta H_{m}$ (150 J/g) was the enthalpy of melting 100% of PVA crystallites. The crystallinity outcomes of PVA samples are presented in Supplementary Table 1.

### Hydrogel Refractive Index Measurement.

A series of hydrogel membranes were prepared via spin coating using a spin coating instrument (SETCAS, KW-4A) on silicon (Si) substrates (University Wafer, Inc., Model 447). The Si substrates were cut into square wafers (13.5 mm x 17.5 mm) using a diamond cutter and then subjected to a rigorous cleaning process. The cleaning process involved washing and ultrasonication in Acetone (Sigma-Aldrich 179124, 99.5%) for 3 minutes, followed by rinsing with MilliQ water. The Si wafers were then washed and ultrasonicated in 30 wt.% H_2_SO_4_ solution (Fisher Chemical 210524, 95.0%) for 3 minutes, followed by rinsing with MilliQ water. Finally, the Si wafers were washed and ultrasonicated in 10 wt.% of H2O2 solution (Sigma-Aldrich 216763, 30 wt.% in water) for 3 minutes, followed by rinsing with 95% ethanol (Fisher Chemical A962P4, 95.0%). The Si wafers were mounted on the spin coater and coated with 10P-GA, 10P-1T-GA, 10P-2T-GA, 10P-3T-GA, and 10P-4T-GA membranes (n=4 for each group) at 1000 rpm for 10s, and at 5000rpm 50s. PVA solutions used for the membranes were prepared using the same method as discussed previously. After spin-coating, the PVA membrane-coated Si wafers were allowed to cross-link and dry in the air for at least 12 hours and then annealed at 100 °C for 20 minutes. The refractive index (RI) of the PVA membrane-coated Si wafers was measured using an ellipsometer (J.A. Woollam RC2) in the range of 400 nm to 700 nm. The measurements were carried out on the membranes in their desiccated states. A series of COMPACT hydrogel membranes (0-4 wt.% TEOS) were prepared using a similar procedure as described above but using a rectangular mold (21.5 × 21.5 × 1 mm). The membranes were demolded after cross-linking, dried at room temperature for 12 hours, and cut into small sheets (2 × 2 mm). The sheets were then annealed at 100 °C for 20 minutes and reswelled in MilliQ water for 1 hour. The RI of the membranes in their hydrated states was measured using a refractometer (Sper Scientific 300034) with water used for calibration.

### Hydrogel Absorbance and Fluorescence Measurement.

A set of hydrogel membranes (designated as 10P-GA, 10P-1T-GA, 10P-2T-GA, 10P-3T-GA, and 10P-4T-GA, comprising 4 replicates for each group) were synthesized and cross-linked in a 96-well plate using established techniques. Subsequently, 1 mL of PVA solution was added to each well and allowed to cross-link and air dry for at least 12 hours, followed by annealing at 100 °C for 20 minutes. Rehydration of the membranes was achieved by the addition of 100 μL of MilliQ water to each well. To obtain transmittance spectra in the range of 400 nm to 700 nm, the 96-well plate was subjected to analysis using a plate reader (Biotek Synergy 2). Autofluorescence measurements were acquired using excitation/emission wavelengths of 470 nm/510 nm and 485 nm/520 nm, respectively. Membrane thickness was determined by caliper measurements and recorded three times to normalize the transmittance spectra and autofluorescence readings with respect to thickness. A blank control consisting of 200 μL of MilliQ water was included for comparison purposes.

### Mechanical characterization of hydrogel fibers.

To ensure consistency, all hydrogel fibers were hydrated prior to the extension test. Tensile tests were conducted using a tensile instrument equipped with a 50N load cell (Stable Micro System TA, XT plusC). The fibers were stretched at a constant rate of 1 mm/second. The nominal stress was calculated from the formula $\sigma =\frac{F}{A}$, where F represents the force measured by the instrument, and A represents the cross-sectional area of the fibers in their hydrated state. The strain was calculated usings $\varepsilon =\frac{\Delta L}{L}$, where ΔL represents the displacement and L represents the initial gauge length. Two marks were labeled on the fibers using a sharpie pen to determine the initial gauge length L prior to the tensile test. A high-resolution camera was used to capture the entire tensile process and track displacement. The stress-strain curve was generated based on the calculated nominal stress and strain. The elastic moduli (E) were determined by calculating the average slope of the stress-strain relationship in the first 10% of applied strain. The average slope was determined by linear regression analysis (OriginLab Corporation). The stretchability of the fibers was reported as a percentage of the strain at the fracture point obtained from the stress-strain curves.

### Light attenuation of hydrogel fibers.

The light transmission loss of hydrogel fibers was tested by the cutback method. Ferrule-connected hydrogel fibers were inserted into a plastic tube (5 cm in length and 3 mm in diameter) and injected with 1 wt.% agar gel to maintain their hydrated state. The ferrule was connected to a 470 nm LED light (Thorlabs M470F3) via an adaptor (Thorlabs SM1FCM). The power (in dB) of transmitted light through the hydrogel fiber was measured using a power meter (Thorlabs, PM16-122). The original power reading was recorded, and a 5 mm interval of cutting was adapted. Starting from the far end of the ferrule, the output power was measured after each cut using a cutter. The attenuation coefficient (α) was calculated using the formula $\alpha =(\frac{10^{4}}{L_{1}-L_{2}})\cdot \log\;(\frac{P_{1}}{P_{2}})$, where $L_{1}$ and $L_{2}$ represent the original and cut lengths of the fiber in meters, respectively. $P_{1}$ and $P_{2}$ are the transmitted power readings before and after the cut, respectively.

### Dimension measurements of hydrogel fibers.

Microscopic images of hydrogel fibers were captured using a bright field mode microscope (AmScope) in MilliQ water. Three distinct regions of each fiber, namely two ends and the middle part, were imaged. The diameter of each fiber was measured using ImageJ software, with nine measurements taken for each fiber. The length of the fibers was measured using a caliper, with three measurements taken for each fiber.

### SEM imaging.

SEM was performed on dried samples using an FEI Magellan 400 XHR instrument. To analyze the cross-sectional morphology of the integrated hydrogel optrode probe, the probe was sectioned into thin pillars (0.1 mm in height) and subsequently mounted on carbon tape for imaging.

### TEM imaging.

The TME images were acquired under a transmission electron microscope (FEI Tecnai 12). The carbon nanotubes were diluted (1:10) in MilliQ water and deposited on a copper grid (Sigma-Aldrich, FCF200-Cu) for imaging.

### Stability tests of hydrogel fibers.

The fabricated COMPACT hydrogel fibers (3 wt.% TEOS) were incubated at 37 °C under physiological-like solutions (saline, ionic strength 305~310 mOsm, pH from 6.0 to 8.0) over 3 months to validate the stability of hydrogel materials. The dimensions of fiber were measured before and after the incubation and statistical analysis was performed on the dimensions between pre-incubation and post-incubation each week.

### Cell culture and biocompatibility tests.

The HEK 293FT cell line was maintained in DMEM (with GlutaMax, Sigma Aldrich, D5796) + 10% fetal bovine serum and seeded in a 24-well plate. COMPACT hydrogel fibers (3 wt.% TEOS) were incubated in DMEM for 24 hours at 37 °C. Hydrogel-incubated DMEM was then added to the well plate and incubated for 24 hours. Calcein-AM (green, 2 μL of 1 mg/mL per well, Sigma-Aldrich 17783) was added to indicate living cells, and ethidium homodimer-1 (red, 2 μL of 1 mg/mL per well, Sigma-Aldrich 46043) was added to indicate dead cells. A fluorescent microscope (Nikon TiU with SOLA Light Engine Gen III illumination hardware and PCO panda sCMOS camera) was used to take images of cells with and without hydrogel incubation. Image J was utilized to count living cells and dead cells. Cell death rate (%) was calculated by using the formula: $\mathrm{death}\;\mathrm{rate}\;(\%)=\frac{\mathrm{dead}\;\mathrm{cell}\;\mathrm{numbers}}{\mathrm{total}\;\mathrm{cell}\;\mathrm{numbers}}\cdot 100\%$.

### Electrochemical impedance spectroscopy (EIS) of COMPACT hydrogel electrodes.

The impedance of COMPACT hydrogel electrodes was assessed using an Electrochemical working station (Princeton Applied Research, PARSTAT 2273) by applying a sinusoidal driving voltage (10 mV, 10 Hz ~1 MHz). Impedance spectra of COMPACT hydrogel electrodes were acquired in PBS solutions.

### Virus package.

pAAV-hSyn-GCaMP6s-WPRE-SV40 was a gift from The Genetically Encoded Neuronal Indicator and Effector Project (GENIE) and D. Kim (Addgene viral preparation no. 100843-AAV9). AAV9- hSyn-GCaMP6s were prepared in Rao Lab at UMass Amherst with Beckman Coulter Ultracentrifuge Optima XL70 with VTi 50.1 rotor. Before use, the viral vector was diluted to a titer of 10^12^ transducing units per milliliter.

### Animals.

All animal surgeries were reviewed and approved by the Committee on Animal Care at the University of Massachusetts Amherst. Wild-type (C57BL/6J) mice and *Thy1::ChR2-EYFP* mice were purchased from the Jackson Laboratory. Mice were given ad libitum access to food and water and were housed at 24 °C ± 1 °C, with 50% relative humidity, and on a 12-h light/12-h dark cycle. All experiments were conducted during the light cycle.

### In vivo hydrogel optical fiber implantation into the mouse brain.

C57BL/6J mice were anesthetized using 1.0% isoflurane administered in a chamber and subsequently secured onto a stereotactic frame (RWD Life Science) with a heating pad to maintain their body temperature. All surgical procedures were conducted in sterile conditions with 1% isoflurane used to maintain anesthesia. The Allen Brain Atlas was used to align the skull and determine the coordinates for viral injection and fiber implantation, specifically targeting the ventral tegmental area (VTA) at coordinates AP: −2.95 mm, ML: ± 0.50 mm, DV: −4.80 mm. An opening was made in the skull using a micro drill (RWD Life Science) at the designated coordinates. A total of 600 nL of adeno-associated virus (AAV) carrying hSyn::GCaMP6s was injected into the target region via a micro syringe and pump (World Precision Instruments, Micro 4). The viral injection device was held in place in the VTA region for 15 minutes to facilitate virus diffusion. Following fiber probe insertion, the probes were lifted by 0.1 mm to accommodate for the viral volume. Finally, the fiber probes were secured to the skull using an adhesive (Parkell, C&B METABOND) and reinforced using dental cement (Jet Set-4). The mice were monitored on the heating pad following removal of isoflurane until they were fully awake.

### In vivo optrode device implantation into the mouse brain.

*Thy1::ChR2-EYFP* mice were anesthetized with 1.0% isoflurane and placed on a stereotactic frame (RWD Life Science) equipped with a heating pad to maintain body temperature. Surgery was conducted under sterile conditions, and 1% isoflurane was continuously administered to maintain anesthesia. Allen Brain Atlas was utilized to align the skull and establish optrode device coordinates (VTA, AP: −3.00 mm, ML: + (or −) 0.45 mm, DV: −4.80 mm) based on the mouse brain atlas. Prior to optrode implantation, a ground screw was implanted (AP: −3.50 mm, ML: − (or +) 1.50 mm, DV: −0.20 mm) and cerebrospinal fluid was contacted with the screw. The optrode devices were fixed on the skull with adhesive (Parkell, C&B METABOND) and reinforced with dental cement (Jet Set-4). Following the removal of isoflurane, the mice were monitored on the heating pad until fully awakened.

### Fiber photometry recording.

Following a four-week recovery period, hSyn::GCaMP6s injected mice were tethered to a fiber photometry (FIP) system using a silica fiber (with a core diameter of 400 μm and a numerical aperture of 0.5, Thorlabs FP400URT). The silica fiber was connected to the FIP system using an adaptor (Thorlabs SM1SMA), and a ferrule (Thorlabs CF440) was fixed to the other end of the fiber. The ferrule was coupled to the implanted fiber probe using a connecting sleeve (Thorlabs ADAF1). The mice were placed in a custom-made chamber (20 × 20 × 20 cm) for social preference tests, and fluorescent signals were computed using custom-written Python code. To excite the fluorescent signal, a custom setup consisting of a 470 nm LED (Thorlabs M470F3), a 405 nm LED (Thorlabs M405F3), and dichroic mirrors (Thorlabs DMLP425R) were used. Illumination periods were determined by detecting synchronization ON/OFF pulses for each LED, with each illumination containing pulses at 10 Hz. To eliminate moving artifacts, the fitted 470 nm signals were subtracted from the fitted 405 nm signals.

### Social behavioral assay.

For all behavioral experiments, adult C57BL/6 mice implanted with optical fiber probes were utilized during the dark phase of the light/dark cycle and were given at least 30 minutes of acclimatization in the behavior chamber before testing. Adult male C57BL/6 mice aged 5-6 weeks were used as strangers, and tests were performed in a dark environment. A chamber box (20 × 20 × 20 cm) containing a social cage was utilized for social interactions. Subsequently, a novel mouse was introduced to the social zone, and the test mouse was exposed to the novel mouse and allowed to interact freely. Concurrently, GCaMP fluorescence changes were recorded during social tests. A dark-vision camera was installed above the social chamber to record video footage during the social tests. The time spent interacting and the distance of social interaction were analyzed using customized algorithms for social interaction assessment with DeepLabCut. The analyzed social interaction epochs were then correlated with GCaMP signals.

### Immunohistology.

The mice were euthanized using fatal plus (Vortech Pharmaceuticals, LTD) and transcardiac perfusion was carried out using 20 mL of PBS (Sigma-Aldrich P3813) solution followed by 20 mL of 4% paraformaldehyde (PFA, Sigma-Aldrich 8187151000) solution. The brains were then dissected from the bodies and fixed in 4% PFA solution at 4 °C overnight. After fixation, the brain tissues were treated with 30% sucrose in PBS for 2 days and subsequently frozen at −20 °C in an O.C.T. cube (21.5 × 21.5 × 22 mm) and sectioned on a cryostat (Leica CM1900) with a thickness of 20 μm. The sectioned tissues were then permeabilized in PBST (0.3% Triton-X-100 in PBS, Sigma-Aldrich 93443) for 15 minutes at room temperature and blocked with 1% bovine serum albumin in PBS (Sigma-Aldrich A9647) for 30 minutes prior to staining. Primary antibody solutions (Iba1 Rabbit and GFAP Rabbit, Agilent Dako, Z0334, at a dilution of 1:400 in PBS) were applied to stain the tissues and incubated overnight at room temperature. After washing the tissues with PBS three times, secondary antibody solutions (GFAP: Thermo Fisher Scientific, Donkey anti-Rabbit IgG (H+L) Highly Cross-Absorbed Secondary Antibody Alexa Fluor 488 Invitrogen, #A-21206; Iba1: Thermo Fisher Scientific, Donkey anti-Rabbit IgG (H+L) Highly Cross-Absorbed Secondary Antibody Alexa Fluor 555, # A-31572; dilution: 1:200 in PBS) were applied and incubated at room temperature for 2 hours. The tissues were then washed with PBS three times and mounted on glass slides. DAPI mounting medium (Southernbiotech, Fluoromount-G, Cat. No. 0100-01) was used to mount the coverglass on top of the glass slide with the sections. The slides were left to dry in air at room temperature overnight before images were acquired using a confocal microscope (Leica SP2).

### Electromyography.

EMG signals were recorded from the gastrocnemius muscle with one reference needle electrode, one hydrogel working electrode (287 ± 14 μm) and one ground electrode. A 473 nm laser (200 mW/mm^2^, 0.5 Hz, pulse width 50ms) was used for transdermal optical stimulation. EMG data triggered by optogenetic activation were collected through a DAM50 system.

### In vivo electrophysiology.

Electrophysiological recordings were performed by connecting the pin connectors of optrode devices to a DAM50 recording system. Optical illumination was carried out using a 473 nm laser connected to the implanted optrode devices via a ferrule-sleeve-ferrule connecting system. The laser (10 mW/mm^2^) was pulsed at a frequency of 0.5 Hz with a pulse width of 50 ms during optical stimulation. Signals were sampled at 50 kHz and filtered between 1-1000 Hz. The amplitude and noise level of evoked potentials were analyzed using a MATLAB algorithm.

## Figures and Tables

**Table.1.** TEOS and PVA concentrations of PVA-TEOS solutions

TEOS: HCl: H_2_O (molar ratio)	TEOS wt.% in PVA pre-solutions	HCl wt.% in solutions	PVA wt.% in solutions
1: 4: 4	2	0.014	10
2: 4: 8	4	0.014	10
3: 4: 12	6	0.014	10
4: 4: 16	8	0.014	10

**Table.2.** TEOS and PVA concentrations in PVA-TEOS-GA fibers

Nomenclatura	TEOS: HCl: H_2_O (molar ratio)	TEOS wt.% in fibers	HCl wt.% in fibers	GA wt.% in fibers	PVA wt.% in fibers
10P-1T-GA	1: 4: 4	1	0.007	0.005	10
10P-2T-GA	2: 4: 8	2	0.007	0.005	10
10P-3T-GA	3: 4: 12	3	0.007	0.005	10
10P-1T-GA	4: 4: 16	4	0.007	0.005	10

## Data Availability

The custom code used in this study is available from the corresponding author upon reasonable request.

## Data availability

The data supporting the findings of this study are available from the corresponding author upon reasonable request.
